# Remarkable Case of Ischuria Renalis

**Published:** 1829-01-01

**Authors:** 


### ISCHURIA RENALIS. DR. BROWN.

This is a formidable complaint at all times. There is probably no excretion which will not bear to be suspended with less inconvenience or danger than the urinary. The following case, which we shall abridge, is interesting, as shewing that ischuria renalis, even in aged persons, is not always a fatal symptom. It is published in Dr. Brown's recent Essays.

Mr. ———, aged 73, was attacked on the morning of the 8th of Jan. 1827, with severe pain in the left iliac region, which recurred at intervals till the 11th, when Dr. B. saw the patient. He had made no water during that time. Blood, exhibiting a strong fibrinous coat, had been drawn by the surgeon in attendance, and the bowels had been freely acted on. There was no pain—no tenderness on pressure over the region of the kidneys—pulse 74, and full. The catheter was introduced, but no urine found in the bladder. Twenty ounces of sizy blood were drawn from the arm—and a catheter was administered. 12th. The same state precisely. No urine discharged — none in the bladder. During last night the patient had a severe paroxysm of pain in the direction of the left ureter. Has thirst, and a salt taste in the mouth. Bowels torpid. Another cathartic. 13th. The patient had severe pain in the iliac regions during the night. General state the same as before. No appearance of urine. Bowels open. Calomel and squills exhibited. Blister to the loins. 14th. No change. A turpentine injection (which had been given before) to be repeated. At 3 o'clock this day, there was a mercurial fetor perceived on the breath—and soon afterwards he discharged about four ounces of water. The mercurial and squills to be continued. 14th. Discharged upwards of five pints of water in the last 24 hours. The mouth a good deal affected by the mercury. The progress of the cure was, from this time, uninterrupted.

*Remarks*. We agree with the highly talented narrator of this case, that the disease was a suspension of the urinary secretion, and not a retention of it in the pelvis of the kidneys. We coincide with Dr. Brown on another point, and that a practical one—namely, that, "the simultaneous occurrence of mercurial fetor on the breath, and restoration of urinary secretion furnished presumptive evidence of the mineral having been beneficial."

Whether the cause of the suspension of secretion was *inflammation* of the kidneys (as Dr. Brown thinks) we shall not presume to determine. The sizy state of the blood, and the pain which was occasionally evinced in the regions of the kidneys and direction of the ureters, give sanction to the opinion; but the discharge of 5½ pints of "*clear pale fluid*," on the first restoration of secretion, is not very characteristic of renal inflammation. The practice pursued in this case, is highly deserving of attention. In the same Essay, there are some other cases, and a dissection, to which we would strongly invite our brethren. Sir Henry Halford, in his very interesting paper on the above subject, has limited to three days the period during which the function of the kidneys can be completely suspended without fatality. In one of Dr. Brown's cases the secretion was suspended for six days and six hours—and in another, for eleven days—yet did the patients recover.

